# Changes in ovarian function in premenopausal women with breast cancer undergoing adjuvant TC (docetaxel and cyclophosphamide) chemotherapy during a brief period of amenorrhea around the last chemotherapy cycle

**DOI:** 10.1186/2193-1801-3-352

**Published:** 2014-07-10

**Authors:** Keiko Yoshimura, Yoshihiko Furuya

**Affiliations:** Department of Surgery, Saiseikai Osaka Nakatsu Hospital, 2-10-39 Shibata, Kita-ku, Osaka, 530-0012 Japan

**Keywords:** Adjuvant treatment, Amenorrhea, Chemotherapy, Early breast cancer

## Abstract

**Purpose:**

Docetaxel, a chemotherapeutic agent, induces high rates of transient chemotherapy-induced amenorrhea (CIA) when used as adjuvant chemotherapy for premenopausal women with breast cancer. Clinical laboratory data to assess the hormonal environment implicated in inducing transient CIA was assessed.

**Methods:**

An observational study was conducted in 35 premenopausal women with hormone-responsive breast cancer who were receiving adjuvant docetaxel/cyclophosphamide (TC) chemotherapy. Serum estradiol and follicular stimulating hormone (FSH) levels were measured at one (n = 6) or two (n = 29) time point(s) around the completion of chemotherapy.

**Results:**

As early as week 6 after the start of chemotherapy, just before the third TC cycle, serum estradiol levels were invariably suppressed (median of 5.5 pg/ml, n = 15, range <5–18.7 pg/ml) and FSH levels increased (median of 63.9 mIU/ml, range 24.5–127.4 mIU/ml), indicative of ovarian suppression to the menopausal levels. Subsequently, at 9 and 12 weeks, serum estradiol levels were suppressed to a median of 6.6 pg/ml (n = 49, range <5–17.3 pg/ml), while FSH levels were high (median of 66.8 mIU/ml, range 29.2–134.5 mIU/ml). There was a significant Spearman’s correlation (ρ = 0.95, n = 29, p < 0.01) of high serum FSH levels (24.5–134.5 mIU/ml) between two time points of repeated measurements in 29 patients. TC chemotherapy induced rapid ovarian suppression with the formation of a high and stable plateau in serum FSH levels from week 6 to week 12.

**Conclusions:**

Recovery from transient CIA post-therapy may be partially attributed to high, stable FSH levels that occurred as early as after completion of the second TC chemotherapy cycle.

## Introduction

Breast cancer is a heterogeneous disease for which optimal treatment must be tailored to a specific breast cancer type (Goldhirsch et al. 2007; Nguyen et al. 2008). Age and menstrual status are taken into consideration when treatment is being planned for patients with early breast cancer. Docetaxel-containing chemotherapy, TC (docetaxel/cyclophosphamide), has been reported to be associated with a better overall survival benefit compared with second generation chemotherapy, AC (doxorubicin/cyclophosphamide), and has become one of the standard adjuvant chemotherapy regimens for the treatment of early breast cancer (Jones et al. 2006; Jones et al. 2009).

Chemotherapy-induced amenorrhea (CIA) varies in type and onset. Amenorrhea was shown to be transient, lasting for 3 (Parulekar et al. 2005; Yoo et al. 2013) or 6 months (Swain & Land 2009; Park et al. 2012; Swain et al. 2010a; Swain et al. 2010b), or persistent. Onset of amenorrhea occurred anywhere from soon after chemotherapy completion to up to 24 months of follow-up. At the 12-month assessment, after either first or second-generation chemotherapy, 41% of patients who were not amenorrheic at the 6-month assessment had CIA (Parulekar et al. 2005). Docetaxel-containing chemotherapy caused amenorrhea at a relatively high rate soon after chemotherapy completion, but at the later periods, 12 and 36 months, the rate of CIA was relatively lower (Berliere et al. 2008; Han et al. 2009). Most (92%) premenopausal women treated with AC followed by docetaxel in the NSABP B-30 clinical trial were reported to become amenorrheic for a short period (<3 months), while 45.3% of women <40 years of age resumed menses at the 2 year assessment (Swain & Land 2009). Anderson *et al.* demonstrated that serum estradiol concentrations decreased at six months only in women who had completed a six-month course of a docetaxel-containing regimen (anthracycline-based chemotherapy followed by docetaxel) (Anderson et al. 2006). It may be possible to determine if there was a brief period of amenorrhea, which the patient might not have recorded even if there was no vaginal bleeding at the time of TC chemotherapy completion, by monitoring ovarian function.

CIA has been associated with clinically important effects on symptoms and quality of life (Knobf 2006). We sought to describe our own experience in this observational study by monitoring serum estradiol and follicle-stimulating hormone (FSH) levels to provide information on changes in serum estradiol levels to deal with the predictable symptoms caused by such hormonal changes, and to gain insight into the underlying hormonal changes due to current CIA.

This study was limited by the small number of patients; however, to the best of our knowledge, this is the first report to show that TC chemotherapy in premenopausal women with hormone-responsive breast cancer induced ovarian suppression at the time of chemotherapy completion, as assessed using clinical laboratory measurements of serum estradiol and FSH.

## Patients and methods

Between May 2009 and March 2014, 35 consecutive premenopausal patients with ER-positive early breast cancer at medium risk who had completed adjuvant TC chemotherapy treatment after surgery were analyzed. Premenopausal status of these patients was defined in this cohort as having regular menses before surgery.

TC chemotherapy consisted of intravenous injections of docetaxel (75 mg/m^2^) and cyclophosphamide (600 mg/m^2^) every three weeks for four cycles (Jones et al. 2006). All 35 patients received complete treatment as defined per protocol. ER and PR status was determined using immunohistochemistry and HER2/neu status was determined using fluorescence *in situ* hybridization (FISH). Tumors were classified as ER-negative when immunohistochemical quantitation showed <1% ER-stained cells. The median age at surgery was 43 years-old and all patients had regular menses before surgery. Their serum estradiol and FSH levels were measured at 6, 9 and 12 weeks after the start of chemotherapy. Patient baseline characteristics are shown in Table 1.

**Table 1:** **Patient baseline characteristics**

		TC group (n=35)
T	1b	4
	1c	16
	2	15
n	0	17
	1-3	18
EIC present		15
Extranodal involvement		1
Histological grade	1	5
	2	21
	3	9
ER	10<	35
	1-9	0
	<1	0
PR	10<	29
	1-9	2
	<1	4
HER2	positive	3
	negative	32

Our original study aim was to monitor changes in serum FSH and estradiol levels three weeks after completion of TC chemotherapy to gather information on baseline levels of the ovarian function, to be compared with levels at the resumption of menses, and all patients gave their consent on this basis. We expanded our study plan to include two time points from earlier cycles of chemotherapy to help pinpoint when estradiol suppression had occurred, because an unexpected stable decline of serum estradiol levels was observed in all patients. Consent to take these additional serum samples was obtained from 29 patients. Fasting morning samples were collected at weeks 6 and 9, just before the start of the third and fourth cycles of chemotherapy, concomitant with regular white blood cell count checks before chemotherapy. Blood samples were also collected from patients not fasting at week 12, three weeks after the forth cycle of TC chemotherapy had been administered.

### Estradiol determination

Serum estradiol levels were determined at BML Inc. (Tokyo, Japan) using a direct immunoassay performed on the Architect-i2000 (Abbott Laboratories, North Chicago, IL), which has an analytic sensitivity of 10 pg/ml, according to the manufacturer’s protocol, with an extended standard curve to allow for a lower limit of detection of 5 pg/ml.

### Statistical methods

The sample size was too small to use an unpaired statistical test; therefore, Spearman’s rank correlation coefficient (ρ) was calculated using repeated measurements of serum FSH levels in 29 women. From the ρ value, t was obtained.

## Results

**Figure 1:**
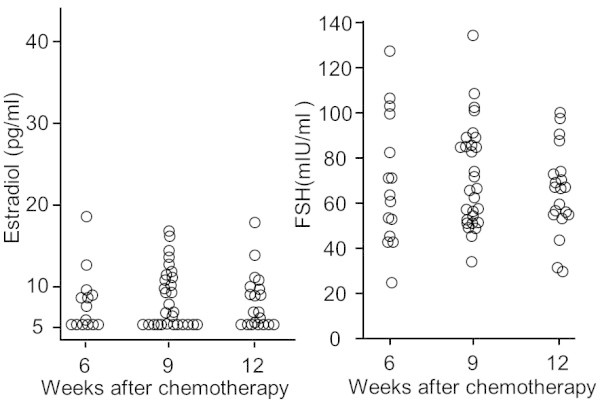
**Serum FSH and estradiol levels following the second, third or fourth cycles of TC chemotherapy in 35 premenopausal women.**

Serum FSH levels above 40 mIU/ml and serum estradiol levels below 39 pg/ml were commonly accepted as baseline values indicating menopausal status. Figure 1 shows serum estradiol and FSH levels from 35 patients at 6, 9 and 12 weeks after the first cycle that TC chemotherapy was administered. The final 15 patients (ages 32–50, median 44 years old) were monitored at two time points, weeks 6 and 9 after the first cycle of chemotherapy.

**Figure 2:**
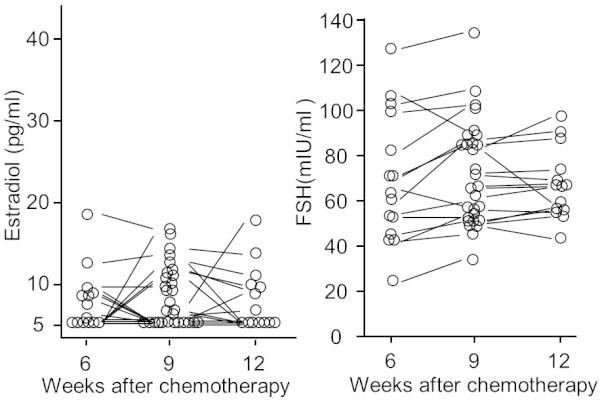
**Bars show inter-individual changes in serum estradiol and FSH levels in later 29 patients who were monitored 2 time-points.**

Serum estradiol levels decreased to below 18.7 pg/ml (median 5.5 pg/ml, range <5–18.7 pg/ml, n = 15) and FSH levels increased to a median of 63.9 mIU/ml (24.5–127.4 mIU/ml) as early as week 6, after two cycles of chemotherapy. At week 9, serum estradiol levels decreased to a median of 6.6 pg/ml (range <5–16.9 pg/ml, n = 29) and serum FSH levels increased to a median of 66.8 mIU/ml (range 33.9–134.5 mIU/ml). Furthermore, at week 12, 3 weeks after administration of the forth cycle of TC chemotherapy, estradiol levels remained decreased with a median value of 6.6 pg/ml (range <5–17.3 pg/ml, n = 20) and FSH levels were still increased with a median value of 66.1 mIU/ml (range 29.2–101 mIU/ml).While Figure 1 indicated that TC chemotherapy resulted in rapid ovarian suppression, the major obstacle to find a statistically significant difference was the small number of patients in the study. In addition, the method used to measure serum estradiol levels had limited sensitivity with a lower limit of detection of 5 pg/ml. Figure 2 shows inter-individual changes in serum estradiol and FSH levels at weeks 6, 9 and 12 in 29 patients with two time point assessments. There was a significant Spearman’s rank correlation coefficient (ρ = 0.95, t = 15.3, n = 29, p < 0.01) between 6 and 9 weeks and/or between 9 and 12 weeks of high serum FSH levels (43.5–102.9 mIU/ml). FSH and estradiol levels fluctuated depending on the phase of the menstrual cycle; therefore, a significant correlation in high serum FSH levels between each of the two time points indicated that these FSH levels may be the maximum that can occur in response to rapid estradiol suppression during the early period of TC chemotherapy.

### Menstruation

At the 2-year assessment, five women (ages 32–48, median 39) resumed menses, confirmed by laboratory measurements, among 20 patients who received tamoxifen only without LHRH analog treatment (ages 32–52, median 45) after chemotherapy completion, with the fastest occurring at week 21. Discordance between laboratory data and patient-recorded vaginal bleeding was observed in two patients who had menstrual bleeding at week 7 among 15 patients whose laboratory data showed severe ovarian dysfunction at week 6.

## Discussion

This study included a small cohort of 35 patients; however, our findings that ovarian suppression at the time of chemotherapy completion was induced in patients treated with docetaxel, were consistent with and corroborated by previous observations in which docetaxel resulted in higher rates of transient CIA when used as adjuvant chemotherapy for premenopausal women with breast cancer (Berliere et al. 2008; Han et al. 2009).

It has been reported that cytotoxic agents, especially anthracyclines and alkylating agents such as cyclophosphamide, induce amenorrhea, potentially by activating apoptotic oocyte death in primordial follicles, which damages the ovarian reserve (Oktem & Oktay 2007). However, younger patients were found to be resistant to CIA because they have a larger primordial follicle pool (Oktem & Oktay 2007). In studies of ovarian function after chemotherapy without docetaxel, the decrease in serum estradiol concentrations and increase in FSH levels showed greater variations than our observations (Yoo et al. 2013; Yu et al. 2010).

Mechanisms of gonadal toxicity caused by chemotherapeutic agents are complex. Colleoni *et al.* reported that three cycles of dose-intensive epirubicin and cyclophosphamide with filgrastim and progenitor cell support significantly improved DFS only in patients with ER-positive disease, and resulted in higher rates of CIA even in patients <40 years old. Their CIA criterion for premenopausal patients was no menses during months 7–9 from randomization (Colleoni et al. 2009). Amenorrhea induced by modern-day chemotherapies appears to be characterized by rapid occurrence and is age-non-specific and transient, contrary to the features of first generation chemotherapy, such as CMF-induced amenorrhea, which occurs at an increasing rate with the passage of time, shows resistance in younger-aged patients, and is permanent.

Docetaxel, given after anthracycline-containing regimens, induced muscle and joint pain in 53–87% of patients (Saibil et al. 2010; Eckhoff et al. 2011). Taxanes (paclitaxel and docetaxel) can induce arthralgia and myalgia in up to 75% of patients (Nugalieva et al. 2010). There are several differences in the toxicity profiles of docetaxel and paclitaxel. Paclitaxel can induce disabling but transient arthralgia and myalgia, called acute pain syndrome, which has been hypothesized to be caused by neurologic injury (Wolf et al. 2008). The incidence of docetaxel-induced peripheral neuropathy is much lower than that of paclitaxel-induced peripheral neuropathy and is usually mild, disappearing spontaneously after discontinuation of therapy (Quasthoff & Hartung 2009; Balayssac et al. 2011).

Symptoms caused by a rapid decline in estrogen levels induced by docetaxel treatment might be overlooked because of the diversity of symptoms and the complexity associated with neuromuscular assessment. There are a few reports that have explored the relationship between pain and rapid decline in estrogens such as that caused by surgical menopause (Fang et al. 2009), but low estrogen levels can lead to an increase in aches and pains. Many women first report joint pain when they are perimenopausal or approaching menopause, though whether aches and joint pains are associated with hormonal changes due to menopausal transition is controversial. The data presented in this study provide a novel aspect of transient CIA by measuring serum estradiol and FSH levels during and after completion of docetaxel-containing chemotherapy, which can be used to track the impact of adherence to treatment, especially for younger patients whose biological changes are more variable than those in patients who are approaching menopause.

Despite the limitations of our study, which was a small single-center pilot study, our findings demonstrated that docetaxel induced a brief period of amenorrhea close to the last cycle of chemotherapy in premenopausal ER-positive breast cancer patients. To understand the hormonal environment and its relationship with a patient’s state as well as symptoms caused by docetaxel-containing chemotherapy, more time points to monitor ovarian function and more patients are needed. Further study is warranted.
